# Non-invasive Vagal Nerve Stimulation Effects on Hyperarousal and Autonomic State in Patients with Posttraumatic Stress Disorder and History of Mild Traumatic Brain Injury: Preliminary Evidence

**DOI:** 10.3389/fmed.2017.00124

**Published:** 2017-07-31

**Authors:** Damon G. Lamb, Eric C. Porges, Greg F. Lewis, John B. Williamson

**Affiliations:** ^1^Brain Rehabilitation Research Center, Malcom Randall VAMC, Gainesville, FL, United States; ^2^Center for Cognitive Aging and Memory, College of Medicine, University of Florida, Gainesville, FL, United States; ^3^Center for Neuropsychological Studies, Department of Neurology, College of Medicine, University of Florida, Gainesville, FL, United States; ^4^Department of Clinical and Health Psychology, College of Public Health and Health Professions, University of Florida, Gainesville, FL, United States; ^5^Department of Psychiatry, School of Medicine, University of North Carolina at Chapel Hill, Chapel Hill, NC, United States; ^6^School of Informatics and Computing, Intelligent Systems Engineering, Indiana University, Bloomington, IN, United States; ^7^The Kinsey Institute, Indiana University, Bloomington, IN, United States

**Keywords:** posttraumatic stress disorder, traumatic brain injury, hyperarousal, autonomic, transcutaneous vagal nerve stimulation, vagal, transcutaneous, sympathetic

## Abstract

Posttraumatic stress disorder (PTSD) is a reaction to trauma that results in a chronic perception of threat, precipitating mobilization of the autonomic nervous system, and may be reflected by chronic disinhibition of limbic structures. A common injury preceding PTSD in veterans is mild traumatic brain injury (mTBI). This may be due to the vulnerability of white matter in these networks and such damage may affect treatment response. We evaluated transcutaneous vagal nerve stimulation (tVNS), a non-invasive, low-risk approach that may alter the functions of the limbo-cortical and peripheral networks underlying the hyperarousal component of PTSD and thus improve patient health and well-being. In this single visit pilot study evaluating the impact of tVNS in 22 combat veterans, we used a between-subjects design in people with either PTSD with preceding mTBI or healthy controls. Participants were randomized into stimulation or sham groups and completed a posturally modulated autonomic assessment and emotionally modulated startle paradigm. The primary measures used were respiratory sinus arrhythmia (high-frequency heart rate variability) during a tilt-table procedure derived from an electrocardiogram, and skin conductance changes in response to acoustic startle while viewing emotional images (International Affective Picture System). The stimulation was well tolerated and resulted in improvements in vagal tone and moderation of autonomic response to startle, consistent with modulation of autonomic state and response to stress in this population. Our results suggest that tVNS affects systems underlying emotional dysregulation in this population and, therefore, should be further evaluated and developed as a potential treatment tool for these patients.

## Introduction

Posttraumatic stress disorder (PTSD) is a common mental illness affecting military veterans (1). Even in transient cases, recurrence can occur in older age. In Vietnam veterans, 10% experienced recurrence of PTSD symptoms nearly three decades after their worst trauma (2). Chronic emotional dysregulation is associated with impairment in quality of life as well as early onset of cognitive decline and serious health consequences (3–6). A common preceding comorbidity is mild traumatic brain injury (mTBI). The etiological contribution of mTBI to manifestation of symptoms of PTSD is not known. However, disruption of limbic white matter may play a role (3), as has been reported in neuroepidemiological studies of the phenomenology of white matter injury in mTBI (7, 8). These systems are directly germane to shifts in emotional and autonomic state to a vigilant and mobilized disposition.

A core deficit in PTSD is a bias toward a defensive strategy to environmental features along with an inability to shift away from a defensive state (3). This defensive strategy manifests in part as hyperarousal features including decreased respiratory sinus arrhythmia [RSA, high frequency heart rate variability (HRV)] and increased sympathetic nervous system response to stressors. In order to shift from defensive dispositions to socially engaging dispositions (e.g., interacting positively with other people), the individual needs to determine safety and inhibit limbic structures that control flight, flight, or freeze behaviors. PTSD is associated with chronic disinhibition of limbic structures such as the amygdala, which may explain symptoms such as exaggerated startle and autonomic nervous system (ANS) mobilization. Because mTBI may effect white matter and, in particular, limbic white matter inputs (7), there may be increased neurological vulnerability to development of symptoms of PTSD and of emotional dysregulation. Though PTSD is a transient condition in many patients, factors such as preceding mTBI may increase the likelihood of a chronic presentation.

The chronic stress associated with PTSD is a critical health issue as the physiological reaction to threat detection is clinically costly. Patients with PTSD have reduced HRV in response to trauma cues, require a longer recovery time (9), and have higher blood pressure (10) than their non-PTSD peers. Several features in resting autonomic behavior are correlated with mortality (11, 12). Reduced resting low frequency HRV is linked to coronary artery disease (13), and lower nighttime RSA is linked to increased stroke risk (14). Further, HRV in patients who recover from PTSD is indistinguishable from healthy controls (15). This suggests that negative health consequences of PTSD may be reversible if treatment success is achieved prior to cumulative damage from chronic stress.

Comorbidities are frequent in patients with PTSD, possibly due to issues with diagnostic clarity [see the recently revised DSM-V (16)], but also because trauma-induced responses are unpredictable and may represent a continuum of defensive state modulation. This produces behaviors consistent with fight, flight, or immobilization that fluctuate depending on internal state and interactions with perceived threat (17–19). Variations in these defensive response styles may, for example, present as depression (immobilization), intermittent explosive disorder (fight), or anxiety (flight).

Many currently available treatments target these states or associated disrupted emotional systems; however, the degree of effectiveness is variable. For example, a common first line pharmacotherapy approach is to use selective serotonin reuptake inhibitors. Unfortunately, clinical response rates, defined as a >30% reduction in symptoms, are rarely over 60%, and fewer than 20–30% of patients with PTSD taking these medications achieve full remission (20–23). Further, although a double-blinded placebo-controlled trial of venlafaxine, a serotonin–norepinephrine reuptake inhibitor, achieved a 78% clinical response rate, only 40% achieved remission (24). Several psychotherapies have been demonstrated to be helpful; however, the effect size varies significantly between reports, and combinations of therapy approaches were more effective in many, but not all, studies (23, 25–28), thus novel approaches are needed to treat those who do not achieve remission with current treatments.

Transcutaneous vagal nerve stimulation (tVNS) is a non-invasive nerve stimulation technique in which the auricular branch of the vagus is targeted. tVNS has been shown to have an impact on the neuronal systems that are involved in emotional regulation (29, 30), including the amygdala, and should be effective in the treatment and rehabilitation of PTSD. In addition, tVNS has been demonstrated to have a low risk profile, which is a significant departure from implanted vagal nerve stimulation (31, 32).

The putative mechanism of action of tVNS is through activation of the nucleus tractus solitarius, which has widespread projections throughout key brain networks involved in emotional regulation and PTSD, and the locus coeruleus. Published pilot fMRI studies have reported BOLD signal alterations in both the nucleus tractus solitarius and the locus coeruleus in the brain stem as well in amygdala activity in response to tVNS as contrasted to a sham stimulation (30). These fMRI changes were observed in healthy individuals who were given tVNS or active sham stimulation. The specific sham or contrasted stimulation varies between studies depending on the specific hypotheses tested; however, findings have generally been consistent. These data support the proposed mechanistic hypothesis, as a reduction in the BOLD signal in emotional (limbic) brain networks should correspond to diminished emotional reactivity and increased socially adaptive emotional regulation (inhibition of fight or flight behaviors), and these same regions and networks are abnormally active in response to emotional stimuli in individuals with PTSD.

Alteration of nucleus tractus solitarius activity should increase high frequency HRV, which has been demonstrated with tVNS in healthy controls (33). Increased high frequency (0.15–0.4 Hz) HRV (RSA) is associated with improved social function, better health outcomes, and better cognitive function. Conversely, lower RSA is associated with many psychiatric and psychological disorders including major depressive disorder, generalized anxiety disorder, high levels of aggression, and trauma history (34–36). tVNS has been shown in healthy people to induce an increase in high frequency HRV for at least 15 min after tVNS use without appreciably altering mean heart rate and other cardiovascular safety measures (32). The latter is important, as it further speaks to the relative safety of tVNS when compared with its implanted counterpart (31). It is unknown whether tVNS has these same positive effects in patients with PTSD or those with disruptions in fronto-limbic function and further, whether tVNS changes emotionally modulated autonomic response.

Because of the high rates of PTSD, particularly subsequent to mTBI, combined with the large proportion of patients who do not achieve clinical response, let alone remission, there is a need for novel treatment approaches. Given the reported impact of tVNS on many brain regions implicated in the development and expression of PTSD, as well as autonomic state, it is a logical tool to develop to potentially treat PTSD. As an initial step toward that end, we have designed the current study to evaluate the impact of tVNS on indices of hyperarousal including vagal tone, as measured by high frequency HRV, and sympathetic nervous system activity in response to emotionally modulated startle as measured by electrodermal activity. We hypothesized that vagal tone would increase and that emotionally modulated sympathetic nervous system activity would attenuate in response to tVNS.

## Materials and Methods

### Subjects

We recruited and received informed consent from 22 combat Veterans to participate in a U.S. Department of veterans affairs (VA) funded, University of Florida IRB approved pilot study designed to assess the effects of tVNS on autonomic symptoms of PTSD. Participants were recruited from a study designed to evaluate the impact of white matter damage in mTBI on the manifestation of PTSD cluster symptoms. Participants were contacted randomly, and three people declined participation due to logistical issues. Participants either had been diagnosed with both PTSD and closed-head mTBI injury (*n* = 12) or were healthy combat controls with no diagnosis of either (*n* = 10). At time of participation, average age was 29.7 years (SD 7) for the healthy combat control group and 30.4 years (SD 5.4) for the PTSD and mTBI group; minimum age was 22, maximum 43. Diagnosis status of mTBI and PTSD were verified *via* a clinical consensus conference using established criteria for each category using VA and DOD diagnostics guidelines. mTBI was defined as an injury to the head as a result of blunt or blast injury with any period of observed or self-reported transient confusion, disorientation or impaired consciousness, dysfunction of memory immediately before or after the time of injury, loss of consciousness less than 30 min, and signs of neurological or neuropsychological dysfunction identified soon after the injury. PTSD status was determined *via* a structured interview and electronic medical record review (VA PTSD clinical evaluations) and self report scales including the PCLD checklist—military (PCL-M), and Symptom Checklist 90—Revised (see Table 1). We also calculated symptom domains by aggregating PCL-M items, with re-experiencing (items 1–5), avoidance (6–7), dysphoria (8–15), and hyperarousal (16–17) as suggested by Pietrzak et al. (37). Mean PCL-M total scores were 24.6 ± 4.83 and 28.0 ± 2.94 (*p* = 0.23) for the healthy combat controls in the tVNS and sham groups, respectively, and 48.5 ± 4.43 and 42.0 ± 12.3 (*p* = 0.31) in the PTSD and mTBI group for the tVNS and sham groups, respectively. The average time since diagnosis of PTSD was 3.55 years, with a range of 1 month to 9 years, although symptom history typically began several years prior to diagnosis after the experienced trauma. One apparently healthy control was excluded upon *post hoc* review of their medical record for sickle cell anemia and one mTBI/PTSD subject was excluded for not having a DOD or VA reported history of mTBI.

**Table 1:** SCL-90-R, BDI-II, and PCLD checklist—military (PCL-M) symptoms per group, mean ± SD, and two sample *t*-test *p*-value.

	Healthy control	Posttraumatic stress disorder/mild traumatic brain injury	*p*
**SCL-90-R scale**			
Somatization	0.43 ± 0.43	0.89 ± 0.74	0.101
Obsessive–compulsive	0.91 ± 0.58	1.71 ± 0.76	0.016
Interpersonal sensitivity	0.54 ± 0.45	1.07 ± 0.48	0.022
Depression	0.54 ± 0.32	1.22 ± 0.75	0.016
Anxiety	0.41 ± 0.29	1.05 ± 0.69	0.016
Hostility	0.61 ± 0.56	1.38 ± 0.8	0.021
Phobic anxiety	0.14 ± 0.23	0.82 ± 0.64	0.006
Paranoid ideation	0.67 ± 0.43	1.14 ± 0.48	0.032
Psychoticism	0.23 ± 0.24	0.7 ± 0.5	0.160
Global severity index	0.51 ± 0.28	1.12 ± 0.56	0.007
Positive symptom distress index	1.45 ± 0.31	1.72 ± 0.37	0.092
Positive symptom total	30.89 ± 15.59	55.91 ± 20.64	0.006
BDI_II	8.22 ± 4.44	18.18 ± 10.85	0.015
**PCL-M**			
Disturbing memories	1.33 ± 0.5	2.8 ± 0.79	<0.001
Disturbing dreams	1.11 ± 0.33	2.8 ± 1.03	<0.001
Re-experiencing	1.11 ± 0.33	1.7 ± 0.67	0.029
Upset when reminded of experience(s)	1.67 ± 0.71	2.4 ± 0.7	0.037
Physical reactions when reminded of experience(s)	1.22 ± 0.44	2.4 ± 0.97	0.004
Avoid thinking or talking about experience(s)	1.33 ± 0.71	3 ± 1.25	0.003
Avoid activities or talking about experience(s)	1.56 ± 0.73	2.8 ± 1.23	0.016
Trouble remembering experience(s)	1.33 ± 0.5	2.1 ± 1.1	0.069
Loss of interest	1.56 ± 0.73	2.8 ± 1.4	0.027
Feeling distant or cut off	1.56 ± 0.73	3 ± 1.49	0.017
Feeling emotionally numb	1.22 ± 0.67	2.7 ± 1.25	0.006
Feeling future will be cut short	1.11 ± 0.33	1.9 ± 1.2	0.072
Difficulty sleeping	1.89 ± 0.93	3.3 ± 0.82	0.003
Irritability or angry outbursts	1.89 ± 0.93	2.5 ± 1.27	0.245
Difficulty concentrating	2.11 ± 0.78	2.8 ± 1.23	0.161
Hyperarousal (alert or on guard)	2.11 ± 1.05	2.9 ± 0.88	0.097
Feeling jumpy or easily startled	2.11 ± 1.17	2.8 ± 1.14	0.210
PCL-M total	26.11 ± 4.26	44.6 ± 10.83	<0.001
**PCL-M aggregate symptom domains**			
Re-experiencing	1.29 ± 0.20	2.42 ± 0.53	<0.001
Avoidance	1.44 ± 0.68	2.90 ± 1.22	0.006
Dysphoria	1.58 ± 0.27	2.63 ± 0.80	0.002
Hyperarousal	2.11 ± 1.08	2.85 ± 0.91	0.13

Exclusion criteria were: premorbid severe psychiatric disorders, other neurological disorder, traumatic brain injury of greater severity than mild (e.g., open-head TBI; loss of consciousness greater than 30 min), medications, which affect ANS responses (e.g., β blockers such as propranolol), and current substance abuse. Assessment of medical exclusion/inclusion criteria was achieved *via* both consensus conference review of VA and DoD medical records and self-report during a structured interview as part of this study. In the healthy combat control group, some participants had recently (within 2 weeks) taken ibuprofen, omeprazole, hormonal birth control, clindamycin, and adalimumab (for colitis/Crohn’s). In the participants with mTBI and PTSD, most (7) were not currently medicated; one was taking simvastatin, trazodone and gabapentin at night, and omeprazole; one venlafaxine, gemfibrozil, ibuprofen, docusate, and trazodone and prazosin at night; one each were taking mirtazapine and paroxitine. Trazadone and prazosin have α-adrenergic pharmacological impacts, but minimal/no β-adrenergic activity, and testing was conducted several half-life durations after most recent dose. Mirtazapine antagonizes α_2A_ and α_2A_ receptors, and to a much lesser extent α_1_, but not β, so an impact on norepinephrine (NE)-mediated effects of tVNS is not expected. Paroxetine has NE transport inhibition and weak α1 receptor activity, but again not β, so an impact on NE-mediated effects of tVNS is not expected.

### Study Design

Participants were randomized into either tVNS or sham (stimulus calibration only) subgroups and were then given a series of assessments of ANS function including emotionally modulated startle and postural HRV assessments. All participants were fitted with custom tVNS electrodes and had comfort threshold calibration, but stimulus amplitude was set to 0 (sham) or 80% of threshold (tVNS) for the remainder of each participant’s session as pre-assigned by randomization.

### Blinding

A researcher blinded to the stimulus condition conducted the self-report questionnaires, structured interview, and data processing. Stimulus grouping (stim versus sham) was un-blinded for interpretation of statistical analysis. Participants were not informed of their stimulus condition but were informed that, after calibration, the stimulus intensity would be set below the calibration level. The investigator who conducted the calibration and set the stimulus level according group (stim/sham) was a different researcher than those who interviewed participants and conducted data processing.

### Vagal Nerve Stimulation

The tVNS stimulus was a 20 Hz, current controlled, 100 μS, alternating polarity pulse delivered *via* an earpiece custom molded for each participant’s left ear with an Ag/AgCl disk electrode held at the interface of the poster wall of the left external auditory meatus and the posterior face of the left tragus, a convenient location to access the auricular branch of the vagus nerve (38). A return electrode was affixed just anterior to the tragus, minimizing stray currents and constraining stimulation. Prior to calibration, participants were informed that the stimulus would be slowly increased until they reported any discomfort, and that the stimulus intensity would then be reduced to a comfortable level for the remainder of the experiment. Individual sensitivity to tVNS stimulation was evaluated with a structured stepped-ramp protocol, with a brief pause at each step during which the participant was asked what the stimulus felt like to them and if they experienced any discomfort. The stimulation intensity was then set to 80% of threshold or 0%, for stimulus and sham groups, respectively; the mean threshold for comfort was 5.6 mA (range 3–11.3 mA). Discomfort was typically described as a mild buzzing or scratching sensation.

### Postural HRV

**Figure 1:**
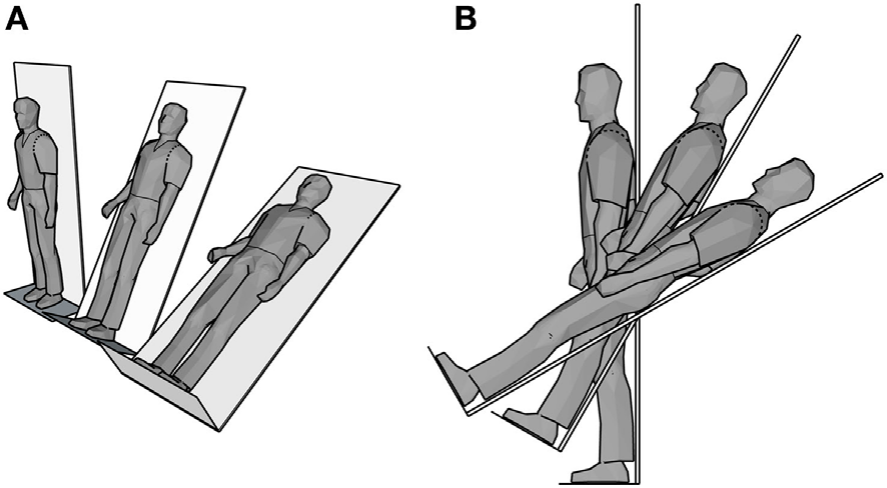
Schematic of postural heart rate variability tilt table test. **(A)** Isometric perspective and **(B)** side view of the three positions (90°, 60°, 30°).

Participants stood with their backs against a motorized tilting table/bed that can be slowly tilted (~2°/s) from 90° (standing) to prescribed angled supine positions (60° and 30°) with the soles of their feet supported at all times by a steel platform (see Figure 1). Continuous blood pressure and electrocardiograms were recorded in 3-min intervals at 90° (standing), 60°, and 30° static table angles. R waves were extracted with a data collection software package (AcqKnowledge 4.1, Biopac systems Inc.) and processed with custom Matlab scripts to correct for missed R-wave detections and apply appropriate filters to extract RSA high frequency HRV. RSA was then analyzed with a mixed effects model with fixed factors for angle and stimulus condition and a random effect of subject in R v3.2.3. One healthy control was dropped from RSA analysis due to having an abnormally low RSA (mean of 2.97, next lowest subject mean RSA was 4.06, overall mean across subjects was 4.99).

### Startle-Blink Paradigm

Participants were given an emotionally modulated startle test while receiving tVNS (or sham stimulation). Participants viewed images from the international affective picture system (39, 40) and were asked to provide an evaluation of the valence (positive—negative) and arousal (neutral/none—high) of each image. During the viewing of these images, an acoustic startle probe (a 50 ms, 95 dB white noise pulse) was delivered during viewing for a predetermined subset of the images. Startle responses, particularly the electrodermal responses (EDA) were recorded and time-synched to the startle probe. Participant’s perceptions of the valence and intensity of affective content were recorded. The resulting electrodermal response (EDA) data were processed with Ledalab V3.4.8, decomposing the signal into tonic and phasic components (41), with analysis epochs triggered on the startle probe onset. Non-responding participants, one sham PTSD/TBI, one tVNS PTSD/TBI, and one tVNS control, identified by no or minimal changes in EDA signal throughout the task, were dropped from analysis; approximately 10% of participants are generally expected to be non-responders (42). We applied a mixed model in R v3.2.3 with fixed effects of group (PTSD/TBI or healthy control) and stimulus (tVNS or sham) and a random effect of subject, with dependent variables measured within each response window of maximum total deflection (phasic and tonic) continuous decomposition (CDA) measure of maximum phasic activity (Phasic Max) and amplitude sum as calculated by Ledalab (41).

## Results

**Figure 2:**
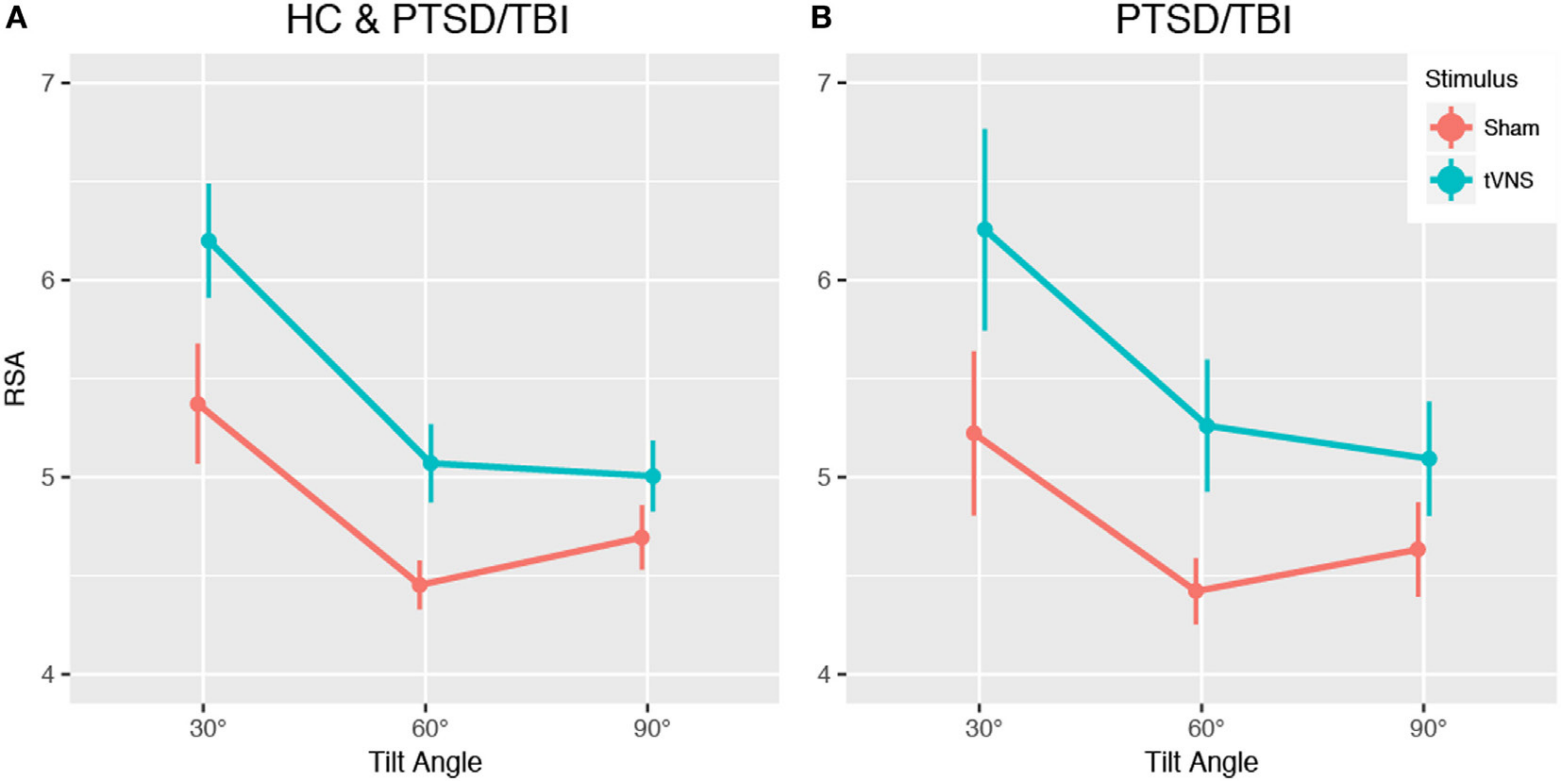
Impact of transcutaneous vagal nerve stimulation (tVNS) on heart rate variability (HRV) during tilt-table experiment. Analysis of pilot data shows a trend toward increased respiratory sinus arrhythmia (RSA) high frequency HRV, indicating increased parasympathetic activity, across all tilt angles in the tilt-table experiment. **(A)** Main effect of tVNS, **(B)** impact of tVNS within PTSD and mTBI group. Data presented are mean ± SEM.

Mean RSA was higher with tVNS than sham across all three postural positions (see Figure 2) indicative of increased parasympathetic activity [tVNS effect, *F*(1, 17) = 3.33, estimated Cohen’s *d* = 0.88]. Diagnosis groups were pooled for this analysis due to insufficient sample size per cell for the full design and the primacy of the question of tVNS efficacy. This finding is consistent with prior reports of increased HRV with tVNS stimulation (32) in healthy populations.

**Figure 3:**
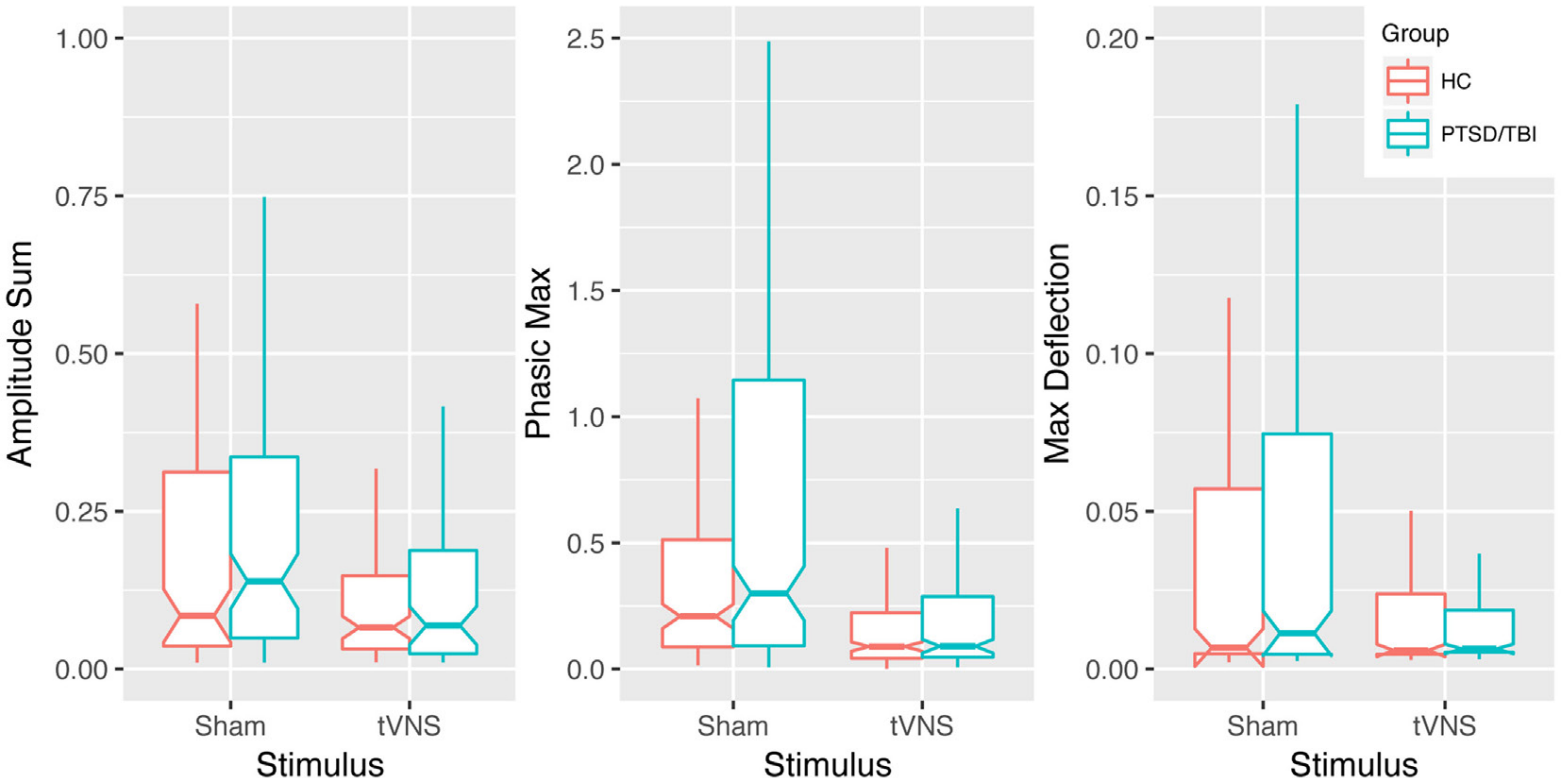
Impact of transcutaneous vagal nerve stimulation (tVNS) on EDA measures. Box plots of phasic max, max deflection, and amplitude sum EDA measures showing a trend of reduced sympathetic activation in response to emotionally modulated startle with tVNS. Notches extend from the median ± 95% confidence interval (1.58/√*n* × interquartile range), whiskers extend from lower and upper quartile to 1.5 × interquartile range.

Transcutaneous vagal nerve stimulation appeared to reduce sympathetic reactivity as measured with EDA and analyzed with continuous decomposition into tonic, or baseline, and phasic activity (41). Our primary concern is with phasic EDA measures maximum deflection within the response window (max deflection) and continuous decomposition analysis phasic (transient response to stimulus) response maximum (phasic max). The estimated effect sizes (Cohen’s *d*) were 0.74, 0.56, and 0.43 for phasic max and max deflection and amplitude sum, respectively (see Table 2 and Figure 3). This is consistent with our anticipated short-term impact of tVNS on emotional/behavioral measures and on RSA.

**Table 2:** EDA reactivity to emotionally modulated startle shows a trend toward reduced reactivity with transcutaneous vagal nerve stimulation (tVNS) in pilot study.

Measure		Estimate	SE	*F*	*d*
Phasic max	Sham—tVNS	0.678	0.419	2.61	0.74
Max deflection	Sham—tVNS	0.114	0.093	1.49	0.56
Amplitude sum	Sham—tVNS	0.163	0.173	0.88078	0.43

## Discussion

The primary findings of this preliminary study are that resting parasympathetic activity is increased and task-dependent emotionally modulated sympathetic nervous system activity is decreased with tVNS. The effect size estimates are promising and the estimated effects are consistent, though it is important to note that the sample sizes are small and full interaction models could not be applied. These were predicted effects based on our model of PTSD with mTBI and the putative mechanisms of action of tVNS (3) and promising reports from animal work using implanted VNS (43, 44). These effects suggest that tVNS may modulate emotional state as reflected by downregulating fight-or-flight and upregulating a physiological state conducive to positive social engagement (45). These results show a direct impact on the hyperarousal symptoms of PTSD by tVNS.

The mechanism of action of tVNS on these systems that are core to the experience of PTSD is not fully elucidated. There are both afferent and efferent components that may be relevant including vagal inputs into the heart and neuroanatomical connections whose activity appear to be modulated by tVNS. Furthermore, VNS can upregulate NE (46). NE is a neuromodulator that plays an important role in the mediation of many behaviors, including emotional learning and attention systems (both critical to the behavior of individuals with PTSD) (47, 48). As with all neuromodulators, NE’s influences on neural function from the cellular level through interacting brain systems level are complex. For example, NE has opposing effects on amygdala network activity through α-NE versus β-NE receptors (49). In the present study, a small subset of participants were prescribed prazosin and/or low-dose trazodone, which have α-adrenergic but not β-adrenergic pharmacology. As these participants took their last dose several half-life durations prior to participation, direct interaction with the NE impacts of tVNS was not expected. One participant was prescribed mirtazapine and another paroxetine, which affect α-adrenergic receptors but not β-adrenergic, so an interaction with the NE impacts of tVNS might have occurred; however, we would expect the most potent attenuation of tVNS to occur with β-adrenergic pharmacology. Instead of avoiding NE pharmacology, as we have done, future studies should consider pharmacologically dissecting the impacts of tVNS with α- and β-adrenergic blocking compounds.

The portions of the frontal lobe that compose networks that interact with the limbic system normally provide inhibition of the autonomic/automatic responses to stimuli previously associated with emotional responses. Consistent with this pattern of inhibition, these portions of the frontal lobes are important in the consolidation of extinction learning (50). In regard to fear learning, there is some evidence that NE-based prophylaxis with β-blockers such as propranolol can prevent the development of PTSD (51). However, this evidence is mixed and complicated by the predominant administration timeline beginning after the traumatic event. β-blocker usage as a treatment adjuvant is also complicated by its impairment of both reconsolidation and extinction learning as well as the attention modulation effects mentioned above.

Prefrontal networks have been strongly implicated in control of the ANS (52, 53). Consistent findings in animals have been found demonstrating a role of the prefrontal cortex in inhibiting sympathetic nervous system mobilization, possibly by modulating parasympathetic action (54, 55) including baroreceptor reflexes (56). Evidence from anatomical, lesion, and electrical stimulation studies suggest that medial prefrontal cortex is preferentially involved in modulating sympathoinhibitory responses, suppressing mobilization of the ANS for fight or flight (57). The full extent of how prefrontal cortex and nuclei involved in autonomic control interact is not known. There are cortical projections to the nucleus of the solitary tract (a major interaction vector for tVNS). These interconnections are involved in blood pressure, vasomotor, and heart rate regulation. In humans, increased heart rate and mean arterial pressure have been associated with decreased regional cerebral blood flow in prefrontal cortex (58).

Transcutaneous vagal nerve stimulation may be considered a parasympathomimetic treatment. It has both direct and indirect potential effects on HRV. Other treatments that manipulate the ANS have shown some promise in alleviating symptoms of PTSD including β-blockers, stellate ganglia blockade, and α channel blockers. Due to the direct impact of tVNS on the underlying brain and autonomic control systems affected by PTSD, tVNS may be a more effective and comprehensive approach to addressing symptoms of PTSD. Core to empirically supported treatments of PTSD are behavioral concepts of extinction and decoupling of a learned threat stimulus from perception of threat and autonomic mobilization. The combination of potential learning system effects (NE), autonomic behavior, and limbic activity may suggest a role of tVNS as a treatment adjuvant.

This study has limitations, particularly the sample size is small. Thus, we are limited in the scope of the statistical models we can apply, so, we cannot analyze a full suite of predictors of individual differences in response. Further, the study was a between-subject, single session design. As such, though there was random assignment to condition, the data are between-subjects and, therefore, could reflect sampling. Finally, our subject blinding technique was psychological and not an active sham, i.e., there was no electrical stimulation in the sham condition. A large N crossover or longitudinal design would likely be more statistically powerful and would also allow for intraindividual comparison; however, the current design does avoid spillover effects and habituation as potential confounds.

## Conclusion

To our knowledge, no studies have been published showing influence of tVNS treatment on alterations of baseline and emotionally modulated autonomic responses in individuals with PTSD. The results of the current preliminary study are promising and should be replicated and extended. What we observed in the current study is a baseline shift in physiological state, i.e., increased markers of parasympathetic nervous system activity. This change in parasympathetic nervous system activity may be interpreted as evidence of a tamping-down of defensive autonomic response and increased amenability to social engagement (45). Further supporting this interpretation, we observed decreased sympathetic nervous system response to emotionally modulated startle. One might conceptualize tVNS as a prosthetic for prefrontal action in inhibiting limbic activity and shifting emotional state to a more socially adaptive form. Autonomic behavior is central to symptoms of PTSD and effective modulation of these systems is associated with better emotional and health outcomes. Thus, further study of tVNS as a potential treatment or adjuvant for patients with emotional dysregulation in the continuum of PTSD is warranted. Follow-up mechanistic work is necessary for delivery impact and optimization and longitudinal effects the symptom clusters of PTSD as well as tolerability and other factors necessary for realization of this tool as a viable treatment approach. In addition to short-term impacts on emotional/autonomic features of PTSD as assessed in the present investigation, tVNS may also have long-term utility with repeated application in reducing symptoms of PTSD.

## Ethics Statement

This study was carried out in accordance with the recommendations of the University of Florida Institutional Review Board with written informed consent from all subjects. All subjects gave written informed consent in accordance with the Declaration of Helsinki. The protocol was approved by the University of Florida Institutional Review Board.

## Author Contributions

JW—conception of project, protocol creation, participant screening, data analysis, writing. DL—conception of project, protocol creation, execution of protocol with participants, data analysis, writing. EP—conception of project, protocol creation, data analysis (quantifying EDA), writing. GL—data analysis (ECG), writing.

## Conflict of Interest Statement

The authors declare that the research was conducted in the absence of any commercial or financial relationships that could be construed as a potential conflict of interest.
